# The Effects of Modified Intermittent Fasting in Psoriasis (MANGO): Protocol for a Two-Arm Pilot Randomized Controlled Open Cross-over Study

**DOI:** 10.2196/26405

**Published:** 2022-02-23

**Authors:** Lynda Grine, Niels Hilhorst, Nathalie Michels, Souheila Abbeddou, Stefaan De Henauw, Jo Lambert

**Affiliations:** 1 Dermatology Research Unit Department of Head and Skin Ghent University Ghent Belgium; 2 Department of Dermatology Ghent University Hospital Ghent Belgium; 3 Department of Public Health and Primary Care Ghent University Ghent Belgium

**Keywords:** psoriasis, leaky gut, gut-skin axis, dietary intervention, intermittent fasting

## Abstract

**Background:**

Psoriasis is a complex disease associated with multiple comorbidities, including metabolic syndrome and leaky gut syndrome. Dietary lifestyle interventions have been reported to affect the disease in terms of lesional severity. It remains unclear how diets affect these comorbidities and the general health in psoriasis patients. Modified intermittent fasting (MIF) on 2 nonconsecutive days has shown beneficial effects on metabolic parameters. A significant advantage of MIF over the currently investigated dietary changes is its feasibility.

**Objective:**

Here, we aim to study the effects of MIF on skin, gut, and metabolic health in psoriasis patients.

**Methods:**

A 2-arm pilot randomized controlled open cross-over study will be performed in 24 patients with psoriasis. Patients will be randomized 1:1 to either start with 12 weeks of MIF and go on a subsequent regular diet for another 12 weeks or start with 12 weeks of regular diet and do subsequent MIF for 12 weeks. The following parameters will be assessed: demographics, disease phenotype, medical and familial history, psoriasis severity, dermatology-specific and general quality of life, nutritional and physical habits, mental and intestinal health, intestinal and cutaneous integrity, inflammatory and metabolic markers, and satisfaction.

**Results:**

A total of 24 participants have been enrolled in the study. The final visit is foreseen for June 2021.

**Conclusions:**

The aim is to uncover the effects of MIF on psoriasis severity and gut health integrity through clinical and molecular investigation. More precisely, we want to map the evolution of the different markers, such as psoriasis severity, permeability, and inflammation, in response to MIF as compared to a regular diet,. Understanding how dietary lifestyles can affect epithelial lineages, such as the skin and gut, will greatly improve our understanding of the development of psoriasis and may offer a nonpharmacological venue for treatments.

**Trial Registration:**

ClinicalTrials.gov NCT04418791; https://clinicaltrials.gov/ct2/show/NCT04418791

**International Registered Report Identifier (IRRID):**

DERR1-10.2196/26405

## Introduction

Psoriasis is a prevalent and chronic skin disease characterized by red, scaly, and thickened skin lesions. The extent of the lesions determines the severity of the disease and is commonly defined by the Psoriasis Area and Severity Index (PASI). The disease has a significant impact on quality of life (QoL) [1]. Currently, no cure is available, and the disease is mainly treated symptomatically.

Psoriasis is a complex and multifactorial disease that remains to be understood more thoroughly. Although genetic factors such as polymorphisms in *PSORS1-9*, *IL12B*, *IL23R*, and *IL28RA* play a role [2], disease development and severity is also heavily affected by factors such as obesity, stress, and smoking [3-11]. Our current understanding of its pathophysiology has led to the development of drugs targeting disease-mediating cytokines such as tumor necrosis factor alpha (TNF-α), interleukin (IL)-17, and the subunits of IL-23 p40 and p19. Interestingly, the same cytokines also play important roles in comorbidities associated with psoriasis [12,13]. Indeed, the disease complexity is evident from the associated physical and mental comorbidities, including cardiovascular diseases, metabolic syndrome, depression, and leaky gut syndrome [14-25]. The latter is especially interesting since it is characterized by an impaired intestinal barrier and hence shows parallels with psoriatic skin, which is also characterized by an impaired cutaneous barrier. The link between the gut and skin has been postulated several times and has been termed the gut-skin axis [26,27]. In the psoriasiform “imiquimod” murine model, we have shown that gut-mediated inflammation drove the extent of the cutaneous lesions and was mediated by the production of type I interferon beta (IFN-β) [28], underscoring that gut health can affect skin health. In humans, intestinal permeability in psoriasis has been reported previously [24,29,30], highlighting the existence of an aberrant gut-skin axis in the disease as well.

The effect of dietary interventions has not been investigated in psoriasis related to this gut-skin axis, as the focus has mainly been on the skin only. Indeed, different studies have been conducted to investigate the effects of diets in psoriasis. For instance, a gluten-free diet was associated with a positive effect on psoriasis severity in patients who tested positive for gluten sensitivity [31,32]. Another study investigating a diet aimed at weight loss showed a favorable outcome on psoriasis and QoL, especially in patients with obesity [33]. This confirmed the findings of an earlier study that combined diet with physical exercise for weight loss [34]. Interestingly, treatment response can be improved by a very low-calorie ketogenic diet [35]. We also reported that treatment response to secukinumab, an IL-17 blocker for psoriasis treatment, may be negatively impacted by weight [36]. Recently, long-term weight loss was found favorable for psoriasis [37]. In 2019, a fasting diet related to Ramadan was conducted in which participants had their meals and drinks, including water, only during evening hours [38]. This diet was found to be favorable for psoriasis, especially in patients treated with apremilast or mTOR inhibitors. More recently, an aggressive ketogenic weight-loss program led to a significant reduction in disease severity in drug-naive psoriasis patients who were overweight or obese [39]. Despite the positive effects of dietary changes on psoriasis outcomes, feasibility for daily implementation and effectiveness heavily depend on adherence. Gibson and Sainsbury propose strategies to increase adherence, including avoiding overcompensation of caloric restrictions and tailoring to the individual’s needs [40]. Here, we aim to investigate the effects of modified intermittent fasting (MIF), more specifically the 5:2 diet. In this diet, participants restrict their caloric uptake to 500 kilocalories (kcal) on 2 nonconsecutive days per week. MIF has been associated with positive outcomes on plasma insulin levels, fat-to-lean ratio, and other cardiovascular disease risk factors [41-44], yet its effects on psoriasis and gut health remains to be investigated. It has a successful adherence rate, as it reduces the drive to overcompensate the calorie restriction and allows the individual to incorporate the calorie-restricting days to their own scheme (tailored), reflecting the conditions postulated by Gibson and Sainsbury for optimal adherence [40].

Here, we present the protocol of the study titled “The Effects of Modified Intermittent Fasting in Psoriasis (MANGO): Protocol for a Two-Arm Pilot Randomized Controlled Open Cross-Over Study” to investigate the effects of a dietary intervention on the gut-skin axis in patients with psoriasis. The MANGO study will provide mechanistic evidence to help determine whether there is a link between gut health and psoriatic lesions, offer insight into the benefit of MIF in psoriasis management, and potentially begin a landmark shift in the holistic view of chronic skin disease.

## Methods

### Research Hypothesis

We aim to investigate the impact of a MIF diet in patients affected by mild psoriasis on skin and gut health based on various markers. The main hypothesis is that a 5:2 diet over the course of 12 weeks will improve skin lesions and gut health biomarkers in comparison to a standard diet.

### Primary Objective

The primary objective of this study is to compare MIF with a standard diet in terms of the proportion of patients obtaining an improvement in absolute PASI score from baseline during or at the end of the 12-week postintervention period to prove the superiority of MIF.

### Secondary Objectives

The secondary objective of this study is to compare MIF with a standard diet during or at the end of the 12-week postintervention period in the following aspects: differences in total body fat, weight, BMI, and waist circumference during and after intervention to baseline; differences in inflammation markers in serum and skin during and after intervention to baseline; differences in metabolic markers in serum and skin during and after intervention to baseline; differences in permeability markers in serum, skin, gut, and feces during and after intervention to baseline; correlation to dietary intake and disease severity; and score of participants’ rating of satisfaction with the intervention.

Finally, the number of participants who complete the study or single intervention window successfully will also be assessed to give us insights into the feasibility of the diet.

### Study Design

**Figure 1:**
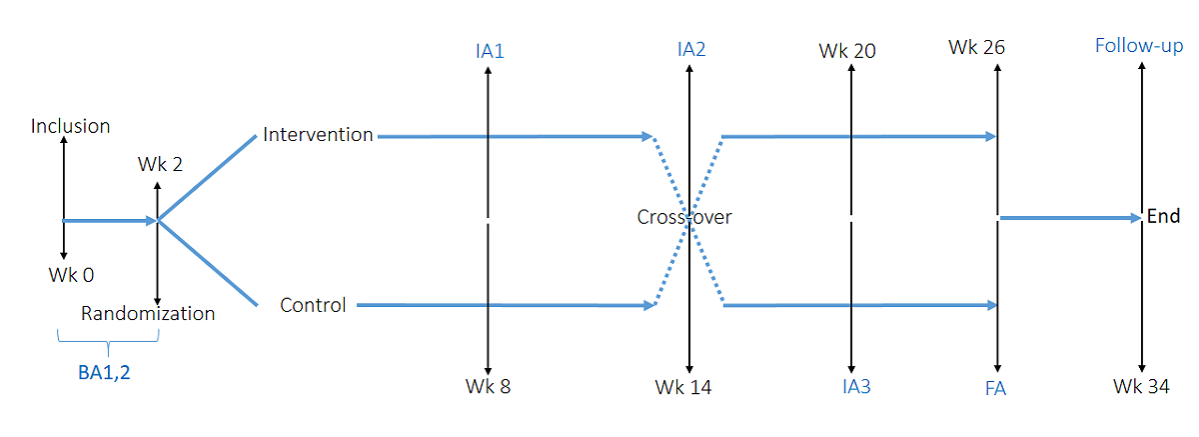
Open randomized controlled cross-over clinical trial to test the effects of modified intermittent fasting on the gut-skin axis in adults with psoriasis. Cross-over includes a 12-week intervention period and a 12-week control period. Evaluations include clinical and biochemical parameters. Intermediate time points are included. BA: baseline analysis; FA: final analysis; IA: intermediate analysis; Wk: week.

We will use an open randomized controlled cross-over clinical trial to test the effects of MIF on the gut-skin axis in 24 adults with psoriasis. The total study duration will be 34 weeks: 2 moments will be included as baseline prior to randomization (week 0 and 2). Randomization will be performed with the REDCap (Vanderbilt University) randomization module upon inclusion. Postrandomization, patients will be assigned to either the intervention or control arm. This study will use a cross-over design, and patients will switch arms at week 14 (Figure 1). Evaluations will include clinical, biochemical, and patient-reported outcomes. Intermediate time points will be included at weeks 8 and 20. Each participant will be in the study for a total of 26 weeks, with a single follow-up at week 34 after completion of the second arm. The entire trial will run for 12 months with a recruitment period of 3 months.

This study has been registered on clinicaltrials.gov (NCT04418791) and has been approved by the ethics committee of the Ghent University Hospital (B6702020000141). The trial will be conducted according to the Declaration of Helsinki. Informed consent will be obtained verbally as well as in writing.

### Recruitment, Eligibility, and Randomization

Study participants are patients who attend the PsoPlus clinic at the Department of Dermatology at the Ghent University Hospital or who are willing to attend the PsoPlus clinic for the study visits. An additional call will be launched through the Flemish Psoriasis League for people with psoriasis to be screened and enrolled. A recruitment target of a maximum of 24 adults will be recruited through consecutive sampling. Participants should have a clinical diagnosis of mild psoriasis vulgaris. Mild psoriasis is defined as a score of 10 or less based on the PASI. The eligibility criteria are further detailed in Textbox 1. Participants who meet any of the exclusion criteria at the time of enrollment or during the study period will be excluded from study participation. Participants will be allocated in a 1:1 manner to the control or intervention arm under stratified randomization with variable permuted blocks, and the concealment of allocation will be based on age, gender, and BMI, with a maximum of 15 participants per arm.

**Eligibility criteria.**

**Inclusion criteria**

- Between 18 and 70 years old
- Clinically diagnosed psoriasis by a dermatologist
- Predominantly present with psoriasis vulgaris
- Present with Psoriasis Area and Severity Index (PASI) ≤ 10 at time of enrollment
- Reported stable weight (< 5% weight loss/gain) for the past 3 months
- Treated exclusively with topical treatment for psoriasis at the time of enrollment and throughout study
- Able to give informed consent
- Willing and able to comply with study procedure
- Willing and able to use MyFitnessPal app to record diet during intervention period
- Willing and able to attend all scheduled visits through the study period
- Willing and able to provide blood, cutaneous, and fecal samples as stated in the procedure
- Willing to apply measures to prevent pregnancy throughout study period

**Exclusion criteria**

- Present with type 1 or 2 diabetes mellitus
- Present with a history of cardiac condition(s)
- Present with comorbidities that cannot be combined with the intervention (eg, cancer)
- History of or current eating disorder (anorexia, bulimia, etc; screening via the *Diagnostic and Statistical Manual Method for Mental Disorders, Fifth Edition* if indicated)
- Malnourished patients (screening via the *Malnutrition Universal Screening Tool* if indicated)
- Present with gout
- Pregnant, having pregnancy plans, or breastfeeding
- Use of diuretics at time of sampling
- Use of anti-, pre-, and/or probiotics in the 3 months prior to enrollment or during the study period

### Study Interventions

Upon inclusion, participants are expected to record their dietary and exercise habits for 2 full weeks using the MyFitnessPal app. Baseline measurements will consist of 2 different time points: inclusion (week 0) and randomization (week 2), which will be averaged for analysis. Upon randomization, participants will be assigned to either the control or intervention arm in a 1:1 ratio. The intervention consists of a dietary intervention based on the 5:2 fasting diet. Participants will perform MIF, for which they will be asked to consume a total of 500 kcal in a window of 6 hours or less from 8:00 AM to 2:00 PM, twice per week on 2 nonconsecutive days. Participants will receive a leaflet with examples of what 500 kcal constitutes. This intervention will last for 12 weeks, starting from randomization. The control arm resembles the baseline period, in which participants can eat without restriction for 12 weeks. A digital food diary will be completed using the MyFitnessPal app, and participants will be asked to use the TARGID tag [45] present in the app’s database. Participants are expected to record their dietary and exercise habits twice a week through the MyFitnessPal app (on the fasting days in the intervention arm). The Food Frequency Questionnaire will be used during all visits to assess and record any dietary changes. Clinical evaluation will be performed during PsoPlus consultations held at the Department of Dermatology at the Ghent University Hospital by the treating dermatologist and specialized nurse. Questionnaires regarding QoL will be completed in the waiting room prior to the consultation, while questionnaires regarding dietary and exercise habits will be completed at home by the patient. Demographic and clinical data will be collected in addition to serum, skin (via tape stripping), feces, MyFitnessPal app, and patient-reported outcomes from questionnaires (Multimedia Appendix 1).

### Outcome Measures

Multimedia Appendix 1 lists the parameters per study visit. During the study, data on demographics will be collected and will include age, gender, and medical and familial history. Furthermore, the disease phenotype will be assessed, and psoriasis severity will be evaluated by an independent assessor. In addition to psoriasis-related parameters, the clinical assessment will include metabolic parameters such as weight, waist circumference, BMI, and total body fat. Lifestyle habits will be registered: patients will keep a diary of their diet twice a week in the online MyFitnessPal app; in the intervention arm, this will be done on the fasting days. General diet and physical exercise habits will be recorded via the Food Frequency Questionnaire and the International Physical Activity Questionnaire, respectively. Questionnaires will also be used to evaluate QoL and mental health, including Dermatology Life Quality Index, EuroQol-5 Dimension-5 Level, Hospital Anxiety and Depression Scale, Beck’s Depression Index, Perceived Stress Scale, and the Visual Analogue Scale for satisfaction.

Cutaneous barrier integrity will be checked through the measurement of transepidermal water loss at 2 different body sites: 1 perilesional and 1 nonlesional site. These will be documented to ensure measurement at the same body sites throughout the study.

Intestinal barrier integrity will be assessed in 2 serological and fecal samples. Permeability markers zonulin, claudin-3, and ileal fatty acid-binding protein (I-FABP) will be quantified in serum, and calprotectin (S100A8/S100A9) will be measured in both serum and stool samples. Participants will also be asked to report on intestinal symptoms based on the Dutch questionnaire for irritable bowel syndrome (Prikkelbaar Darm Syndroom Questionnaire).

Serological levels of inflammatory and metabolic messengers such as IL-6, TNF-α, leptin, and adiponectin will be measured in serum and skin. The latter will be collected through skin tape stripping at the same body sites where transepidermal water loss measurement is performed.

Finally, microbiome sampling will be performed through cutaneous and fecal samples for future follow-up projects.

Study visits will be planned on the fasting days, and participants will be scheduled in this manner to account for circadian rhythm and reduce intraindividual variability.

### Sample Size Calculations

The sample size was determined by power analyses and the available study budget. It was calculated with the power calculator on the Melanoma and Skin Cancer Trial website, with a 2-tailed* t* test and α at .05 and power at 0.80. The predicted effect size was estimated based on a prior study of intermittent fasting [38]. We estimated that at a 2-sided *P* value of .05 and with 80% power, we would need 16 participants to complete the study to detect a within-individual effect size of 0.75 SDs. We estimated a dropout rate of 20%, leading to a sample size of 20 participants. To minimize dropouts, recruiting personnel will emphasize considering the requirements of the study before enrollment begins. In cases of dropouts, extra participants will be recruited to maintain statistical power.

### Statistical Analysis

The primary aim is to explore the effect of MIF on mild psoriasis. Secondary outcomes include changes from baseline to selected time-points during and postintervention in QoL, body weight, BMI, total body fat, inflammation and metabolic markers in serum and skin, and permeability markers in serum and feces. Furthermore, differences in dietary and physical exercise habits will be investigated. Comparisons will be made within a single arm (paired) and between both arms (unpaired).The chi-square test and Mann-Whitney test will be used to compare groups, and regression-binary logistics will be performed with identified independent variables to determine their influence on the outcome. Demographics will be analyzed as confounding variables. Cytokine data that are not normally distributed and differences in cytokine levels between groups will be analyzed using the nonparametric Mann-Whitney test. Correlations will be assessed using Spearman rank correlation. For each group, we will use multivariate logistic regression modeling to detect associations between cytokine levels and the primary end point, and to adjust for confounding effects of age, sex, and the intervention. Cytokine data will be log-transformed prior to use in multivariate models. Pre-existing differences between groups at baseline will be examined using a 1-way analysis of variance featuring a factor for diet allocation. Significant differences emerging from these tests will be explored using appropriate post hoc tests to adjust for multiple comparisons and to isolate the source(s) of variance. In addition to these analyses at the group level, individual responses will also be closely examined for outliers that may affect interpretation. Further analysis, such as subgroup analysis, may also be conducted in light of patterns emerging in the final data set. Baseline characteristics of participants who withdraw during the fasting intervention will also be compared against the final population with t tests being used to assess tolerability. *P* values will be reported to 4 decimal places with *P* values less than .0001 being reported as *P*<.001. A *P* value <.05 will be considered statistically significant. Data analysis will be executed with SPSS 23.0 (IBM Corp) and GraphPad Prism (GraphPad Software Inc).

### Dissemination of Project Findings

The findings of this study will be disseminated by various means, determined by the target audience. To reach the academic dermatology community, we will publish the results in a scientific international peer-reviewed dermatology journal and present our findings at (inter)national congresses with a focus on dermatology and psoriasis. The psoriasis patient community will receive information on the results through the National Psoriasis Foundation and the Flemish Psoriasis League, including a laymen summary of the findings. Lastly, we will reach the general public by communicating the main results through the research team’s social media channels.

## Results

The study initiation was delayed due to the COVID-19 pandemic. Active recruitment of patients began in July 2020, and the first patient was included in October 2020. As of December 2020, we enrolled a total of 24 patients. The last patient visit is foreseen in June 2021, and results are expected to be published December 2021.

## Discussion

Recently, psoriasis has been accepted to be a multimorbid disease with a large impact from lifestyle factors. Obesity has been found to be an independent predictor for the development of psoriasis and to be associated with disease severity [46]. A growing body of evidence suggests the existence of a gut-skin axis, which may also be of importance in psoriasis, revealing the need to urgently address the question of how diet may affect the disease. To our knowledge, the MANGO study is the first comprehensive trial to investigate the effects of MIF on cutaneous, intestinal, and mental health in a cohort of people with psoriasis.

We expect that the MIF intervention will have beneficial effects on psoriatic lesions and be associated with favorable changes in metabolic parameters. We anticipate to detect shifts in intestinal parameters that may be associated with skin improvement. In addition, the data generated from this trial will inform the design of future large-scale trials to evaluate the presence and role of the gut-skin axis in psoriasis. The study additionally includes collection of cutaneous and fecal samples for future microbiome analysis if the intervention proves beneficial.

To overcome the difficulty of diet-based interventions in terms of confounding factors and the small sample size, we have chosen the design of a prospective cross-over randomized trial. As such, each patient will be monitored over more than 6 months and serve as his or her own control. Another strength of this study is the combination of clinical, biochemical, and patient-reported outcomes for cutaneous, intestinal, and mental parameters. Moreover, since some parameters can vary, the study includes 2 baseline points in order to assess normal variation. We opted to perform the pilot trial in a cohort of patients with mild psoriasis who are not on systemic agents to reduce any confounding effects of immunomodulators that may directly impact the outcomes. Lastly, since we introduced a restrictive time window for the consumption of the 500 kcal on fasting days, we will be able to reduce confounding effects of the circadian rhythm [47]. A potential limitation includes health bias among participants.

Results from this study may have multidimensional consequences and assets. On the one hand, beneficial effects of fasting may be potentially viewed as a nonpharmacological add-on treatment for psoriasis. A subset of psoriasis patients dislike pharmacological treatments and therefore opt to gain additional control over their disease through a diet. On the other hand, evidence that a free intervention may have health benefits in patients requiring costly drugs such as biologics may give rise to a moral dilemma: how do we define health responsibility in terms of lifestyle with the rise of health care costs? This debate, applicable to many other (chronic) illnesses, is highly relevant and should not be postponed. It should take place in the near future, with a multidisciplinary panel in a transparent manner in order to interpret the results from comparable trials.

To conclude, if patients with psoriasis tolerate MIF well and experience improvement, there is potential for the diet to be widely adopted by those with psoriasis in a sustainable manner. In addition, MIF may provide a positive impact on their general health, as this diet has already proven to be effective in obesity and seems to be also effective in diabetes [48]—two common comorbidities associated with psoriasis. Given this, we may discern the importance of the gut-skin axis and use it to our advantage in the disease management of psoriasis.

## Appendix

Multimedia Appendix 1 Overview of all parameters collected at each visit divided into clinical, lifestyle-related, and biochemical parameters.

Multimedia Appendix 2 Peer-reviewer report from the National Psoriasis Foundation.

## Abbreviations

I-FABP: ileal fatty acid-binding protein
IL: interleukin
INF: interferon
kcal: kilocalorie
MANGO: Modified Intermittent Fasting in Psoriasis
MIF: modified intermittent fasting
PASI: Psoriasis Area and Severity Index
QoL: quality of life
TNF: tumor necrosis factor
