# Supplementary material for: The Effects of Modified Intermittent Fasting in Psoriasis (MANGO): Protocol for a Two-Arm Pilot Randomized Controlled Open Cross-over Study
Source: JMIR Res Protoc. 2022 Feb 23;11(2):e26405. doi: 10.2196/26405 (PMC8908196; doi:10.2196/26405)
Supplement: Multimedia Appendix 1 [file resprot_v11i2e26405_app1.docx]

| **PARAMETERS** | **STUDY VISIT** | ***Screening*** | ***Visit 1 (BA1)*** | ***Visit 2 (BA2)*** | ***Visit 3 (IA1)*** | ***Visit 4 (IA2)*** | ***Visit 5 (IA3)*** | ***Visit 6 (FA)*** | ***Visit 7 (FU)*** |
| --- | --- | --- | --- | --- | --- | --- | --- | --- | --- |
|  | **TIMING** |  | ***Week 0*** | ***Week 2*** | ***Week 8*** | ***Week 14*** | ***Week 20*** | ***Week 26*** | ***Week 34*** |
| **CLINICAL** | **General** | Gender | Medical history |  |  |  |  |  |  |
|  |  | Age | Familial history |  |  |  |  |  |  |
|  |  | Medical history |  |  |  |  |  |  |  |
|  | **Psoriasis** | PASI | PASI | PASI | PASI | PASI | PASI | PASI | PASI |
|  |  |  | BSA | BSA | BSA | BSA | BSA | BSA | BSA |
|  |  |  | Phenotype |  |  | Phenotype |  |  |  |
|  | **Metabolism** | BMI* | BMI* | BMI* |  | BMI* |  | BMI* | BMI* |
|  |  |  | Waist circumference | Waist circumference |  | Waist circumference |  | Waist circumference | Waist circumference |
|  |  |  | Total body fat | Total body fat |  | Total body fat |  | Total body fat | Total body fat |
|  | **Quality of life** |  | DLQI | DLQI | DLQI | DLQI | DLQI | DLQI | DLQI |
|  |  |  | HADS | HADS | HADS | HADS | HADS | HADS | HADS |
|  |  |  | BDI | BDI | BDI | BDI | BDI | BDI | BDI |
|  |  |  | PSS | PSS | PSS | PSS | PSS | PSS | PSS |
|  |  |  | VAS satisfaction | VAS satisfaction | VAS satisfaction | VAS satisfaction | VAS satisfaction | VAS satisfaction | VAS satisfaction |
|  |  |  | PDSQ | PDSQ | PDSQ | PDSQ | PDSQ | PDSQ | PDSQ |
|  |  |  | EQ-5D-5L | EQ-5D-5L | EQ-5D-5L | EQ-5D-5L | EQ-5D-5L | EQ-5D-5L | EQ-5D-5L |
| **LIFESTYLE** | **Diet** | MUST** | MFP | MFP | MFP | MFP | MFP | MFP |  |
|  |  | DSM-5** | FFQ | FFQ | FFQ | FFQ | FFQ | FFQ |  |
|  | **Physical activity** |  | IPAQ |  |  | IPAQ |  | IPAQ |  |
| **BIOCHEMICAL** | **Blood (serum)** |  | Inflammation markers | Inflammation markers | Inflammation markers | Inflammation markers | Inflammation markers | Inflammation markers |  |
|  |  |  | Metabolic markers | Metabolic markers | Metabolic markers | Metabolic markers | Metabolic markers | Metabolic markers |  |
|  |  |  | Permeability markers | Permeability markers | Permeability markers | Permeability markers | Permeability markers | Permeability markers |  |
|  | **Skin (tape)** |  | Inflammation markers | Inflammation markers | Inflammation markers | Inflammation markers | Inflammation markers | Inflammation markers | TEWL |
|  |  |  | TEWL | TEWL | TEWL | TEWL | TEWL | TEWL |  |
|  |  |  | Microbiome*** | Microbiome*** |  | Microbiome*** |  | Microbiome*** |  |
|  | **Gut (fecal)** |  | Permeability markers | Permeability markers | Permeability markers | Permeability markers | Permeability markers | Permeability markers |  |
|  |  |  | Microbiome*** | Microbiome*** |  | Microbiome*** |  | Microbiome*** |  |
| **BMI based on weight measured with empty bladder**To be used in screening procedure if indicated ***Stored until use in a follow-up project* | | | | | | | | | |
| *Abbreviations: BA: Baseline analysis; BDI: Beck Depression Index; BMI: Body Mass Index; BSA: Body Surface Area; DLQI: Dermatology Life Quality Index; DSM-5: Diagnostic and Statistical Manual of Mental Disorders Fifth edition; FA: Final analysis; FFQ: Food Frequency Questionnaire; FU: Follow-up; HADS: Hospital Anxiety and Depression Scale; IA: Intermediate analysis; IPAQ: International Physical Activity Questionnaire; MFP: MyFitnessPal app; MUST: Malnutrition Universal Screening Tool; PASI: Psoriasis Area Severity Index; PDSQ: Prikkelbaar Darm Syndroom Questionnaire (Irritable Bowel Syndrome Questionnaire); PSS: Perceived Stress Scale; TEWL: Trans Epidermal Water Loss; VAS: Visual Analogue Scale* | | | | | | | | | |
