# Supplementary material for: The Effects of Modified Intermittent Fasting in Psoriasis (MANGO): Protocol for a Two-Arm Pilot Randomized Controlled Open Cross-over Study
Source: JMIR Res Protoc. 2022 Feb 23;11(2):e26405. doi: 10.2196/26405 (PMC8908196; doi:10.2196/26405)
Supplement: Multimedia Appendix 2 [file resprot_v11i2e26405_app2.pdf]

2019 Research Grants

Review Summary

Application Type:
Early Career Research Grant

Principal Investigator:
Lynda Grine, Ph.D.

Project Title:
The gut-skin axis: investigation of dietary intervention in individuals with psoriasis

Outcome:
Funded

Explanation:
In an effort to fund grants that are both scientifically meritorious and aligned with NPF's priorities, NPF conducts a three-stage peer-review process:

Step	Review Activity	Output
1	Preliminary scientific review by two peer-reviewers	Numerical scores and brief rationale
2	Discussion and final assessment by scientific peer-review committee	Numerical scores, discussion notes, and detailed rationale
3	Discussion and assessment by NPF Research Committee	Discussion notes and funding determination

All grants undergo the first step of the review process, but only a fraction move on to the second step, and from there only a fraction move on to the third step. The extent of the comments contained in the following pages of this document depends on the step to which this application advanced. All numerical scores follow the 9-point NIH scoring system:

**High Impact:**	1 – Exceptional	2 – Outstanding	3 – Excellent
**Medium Impact:**	4 – Very Good	5 – Good	6 – Satisfactory
**Low Impact:**	7 – Fair	8 – Marginal	9 - Poor

<u>Reviewer #1:</u>

Relevance to psoriasis and/or psoriatic arthritis: 2

I agree with the investigator that "understanding how dietary lifestyles can affect epithelial lineages such as the skin and gut will greatly improve our understanding of the development of psoriasis and may pose a non-pharmacological venue for treatments".

Ability of investigator to accomplish aims: 3

This is a 26-week prospective cross over trial where patients will undergo a modified intermittent fasting diet vs. regular diet. The overall study is very well designed.

Strategy, methodology, and analyses are well-reasoned and appropriate to accomplish the aims of the project: 6

Although the study is overall well-designed, there are some details of the proposed study that should be addressed. For example, diet will be tracked by a diary kept by the subjects, which is typically unfortunately unreliable. Diet instructions will be provided via "a leaflet containing practical guidance" with a trained nutritionist available. The investigator may consider other methods to track compliance with the restricted diet. Furthermore, no sample size justification or statistical methods are included.

Strength of research environment and mentorship: 4

The investigator has great letters of support and is located in a large medical center with an appropriate patient population. As this is an early career grant, it would be helpful to know more about the mentorship the investigator will receive.

Potential problems, alternative strategies, and benchmarks for success are presented: 3

Limitations and risks, as well as possible solutions, are well-described.

Innovation: 3

No formal studies have been conducted to date to investigate the role of leaky gut and a restricted calorie diet on psoriasis. This is a very hot topic and is of interest to many patients, physicians, and scientists. I commend the investigator for attempting to study a complicated and interesting topic.

Likelihood of leading to additional funding: 3

The study is likely to lead to additional funding for a larger scale/multicenter study.

Likelihood of impacting patients' lives: 2

As patients often ask about how diet affects psoriasis, the findings of this study may help us begin to answer this question. Improvement of psoriasis through dietary measures may also decrease the need for pharmacologic treatment and thus lower the economic burden of psoriasis.

Final Overall Score: 4

This is an exciting study and investigator with a lot of potential. Details of the study design should be ironed out and the mentorship support should be better described.

Reviewer #2:

Relevance to psoriasis and/or psoriatic arthritis: 2

The role of diet in psoriasis is of high interest to patients.

Ability of investigator to accomplish aims: 2

Dr. Grine obtained a PhD in biotechnology in 2014 and certificate for interdisciplinary program in translational medicine in 2018. She is interested in education and co-leads the treat-to-target initiative in Belgium and is interested in identifying biomarkers of disease severity and comorbidities. She has outstanding training and collaborators including a dietician for the project.

Strategy, methodology, and analyses are well-reasoned and appropriate to accomplish the aims of the project: 4

Excellent plan to gather clinical data and biomarkers however it is not clear how many patients are in the trial and no power calculations given.

Strength of research environment and mentorship: 2

Mentor Jo Lambert is Professor Ghent Univ (clinician)
Team includes Dr. Niels Hilhorst (Dietician)

Potential problems, alternative strategies, and benchmarks for success are presented: 3

Adequate plan presented

Innovation: 2

Combining a dietary clinical trial with assessment of biomarkers is innovative.

Likelihood of leading to additional funding: 2

Could lead to larger, randomized clinical trial.

Likelihood of impacting patients' lives: 1

Patients are very interested to know what dietary changes can improve their condition

Final Overall Score: 2

Strengths of this proposal is that it supports the development of a unique investigator with multi-disciplinary background; addresses a topic of high important to patients while linking to more mechanistic parameters; is carefully written so will learn a lot; this is someone we would like to see working in the field of psoriatic research.

<u>Scientific Peer-Review Committee</u>

Final Overall Score: 2.71

Summary of Comments:

Skin-gut connection is a hot topic – of interest to both providers and patients. PI completed PhD in biochemistry and biotechnology. Reviewer 1 was skeptical about how subjects would adhere to 500 calorie diet and how that would be tracked. Reviewer 2 stated that NPF is committed to patient-centered research and clinicians care about diet and how that can affect PsO. PI received certificate in translational outcomes medicine and there is interest in bringing young investigator into field of dermatology. Patients are highly motivated to know if something they eat or don't eat can affect their PsO.

Strengths:
- Hot topic about diet and psoriasis.
- Well laid out proposal.
- Strong investigator and mentor.

Weaknesses:
- Concerns about patient adherence to diet.
- Sample size and statistical methods lacking.
